# Mindfulness-Based Interventions in Recurrent Ovarian Cancer: A Mixed-Methods Feasibility Study

**DOI:** 10.1177/1534735420908341

**Published:** 2020-03-16

**Authors:** Emily Arden-Close, Felicity Mitchell, Gail Davies, Lauren Bell, Carole Fogg, Ruth Tarrant, Roslyn Gibbs, Chit Cheng Yeoh

**Affiliations:** 1 Bournemouth University, Poole, Dorset, UK; 2 Portsmouth Hospitals NHS Trust, Portsmouth, UK; 3 University of Southampton, Southampton, UK; 4 University Hospital Southampton NHS Foundation Trust, Southampton, UK; 5 University of Portsmouth, Portsmouth, UK

**Keywords:** mindfulness, cancer, ovarian cancer, psychotherapy, mental health

## Abstract

A recurrence of cancer is a traumatic and stressful experience, and a number of approaches have been proposed to manage or treat the associated psychological distress. Meditative techniques such as mindfulness may be able to improve an individual’s ability to cope with stressful life events such as cancer diagnosis or treatment. This single-arm mixed-methods study primarily aimed to determine the feasibility of using a mindfulness-based intervention in managing psychosocial distress in recurrent ovarian cancer. Twenty-eight participants took part in a mindfulness-based program, involving six group sessions, each lasting 1.5 hours and delivered at weekly intervals. The study found that the mindfulness-based intervention was acceptable to women with recurrent ovarian cancer and feasible to deliver within a standard cancer care pathway in a UK hospital setting. The results suggested a positive impact on symptoms of depression and anxiety, but further study is needed to explore the effectiveness of the intervention.

## Introduction

Globally, ovarian cancer accounts for approximately 3.5% of all cancer incidence among women.^1^ Some of the highest incidence rates are seen in North America, and North and Eastern Europe. For females in the United Kingdom, ovarian cancer is the sixth most common form of cancer, with around 7,400 new diagnoses each year.^2^ Due to the non-specific symptoms and difficulties in early detection, over 60% of cases of ovarian cancer are diagnosed at an advanced stage,^3^ and in most cases, the disease has already progressed beyond the pelvis. Five-year survival rates are, therefore, low, between 30% and 40% in most countries.^4^ Despite high initial response rates to treatment, over 70% of patients with ovarian cancer will experience chemoresistance and disease recurrence,^5^ and ovarian cancer was the cause of over 184,000 deaths worldwide in 2018.^1^

Experiencing a recurrence of any type of cancer is traumatic and stressful, and it is associated with a high prevalence of concurrent psychological morbidity.^6^ Depression and anxiety are two of the psychological issues most commonly experienced by patients with all cancer types.^7^ Psychological distress can affect a number of cancer outcomes, including quality of life, adherence to treatment, health behaviors, and potentially disease progression and survival,^8^ as well as increase utilization of health care resources.^9^ The National Cancer Survivorship Initiative, launched in the United Kingdom in 2010,^10^ set out to understand the needs of those living with cancer. One of this initiative’s key goals is to improve the management of psychological conditions associated with cancer diagnosis and treatment.

A number of approaches have been proposed to manage or treat the psychological distress associated with cancer. These have included cancer counselling and education, psychotherapies such as cognitive behavioral therapy supportive-expressive group therapy and cognitive-existential therapy, and pharmacotherapies such as antidepressant medication.^11^ There has also been a growing interest in the therapeutic application of mindfulness-based approaches—including mindfulness-based stress reduction^12^ and mindfulness-based cognitive therapy^13^—across a range of health care conditions, including psychological disorders secondary to cancer.

Mindfulness is a meditation practice founded in the traditions of Buddhism. It has been defined as the process of paying attention to the present moment in a non-judgmental manner, and it is proposed to foster clear thinking and openheartedness.^14^ Mindfulness emphasizes the importance of accepting all thoughts and experiences as they are, without trying to alter or change them, and thereby develop a greater sense of well-being. In a clinical context, meditative techniques such as mindfulness may be able to improve an individual’s ability to cope with stressful life events such as cancer diagnosis or treatment. The strongest evidence base is around the use of mindfulness in the treatment of depression and anxiety,^15^ which has led to national guidelines recommending mindfulness-based cognitive therapy for depression in the United Kingdom,^16^ North America,^17,18^ and Australia and New Zealand.^19^ However, there is also further evidence to suggest that mindfulness-based approaches may additionally be effective in combating the psychological distress associated with cancer.^20-23^

A recent meta-analysis by Watts et al found high levels of clinically significant depression and anxiety—25% and 27%, respectively—in patients with ovarian cancer.^24^ Despite the high psychosocial morbidity experienced by these women, there is little research on effective interventions. Most mindfulness intervention studies in cancer so far have focused on patients with breast cancer, a condition with a better prognosis than ovarian cancer due to earlier diagnosis. In contrast, the role of mindfulness-based interventions in managing psychosocial distress in recurrent ovarian cancer is so far unknown.

We undertook a single-arm mixed-methods study to assess the feasibility and acceptability of delivering a mindfulness-based intervention to women with ovarian cancer, which had recurred following initial treatment. The intervention was delivered as a six week program of group sessions, and participants were assessed before and after the intervention and at 3-month follow-up, to aid our preliminary understanding of the effects on both psychological and physiological markers. We also conducted focus groups to qualitatively establish participants’ experiences and perceptions, and to inform the refinement of the intervention for future study and implementation.

## Methods

### Study Design

This single-arm interventional study was conducted at one site in the United Kingdom (Queen Alexandra Hospital in Portsmouth Hospitals NHS Trust), using mixed qualitative and quantitative methods of data collection and analysis. Ethical approval was obtained from the South Central–Berkshire Research Ethics Committee (REC Reference 16/SC/0415).

### Participants and Procedures

**Figure 1.**
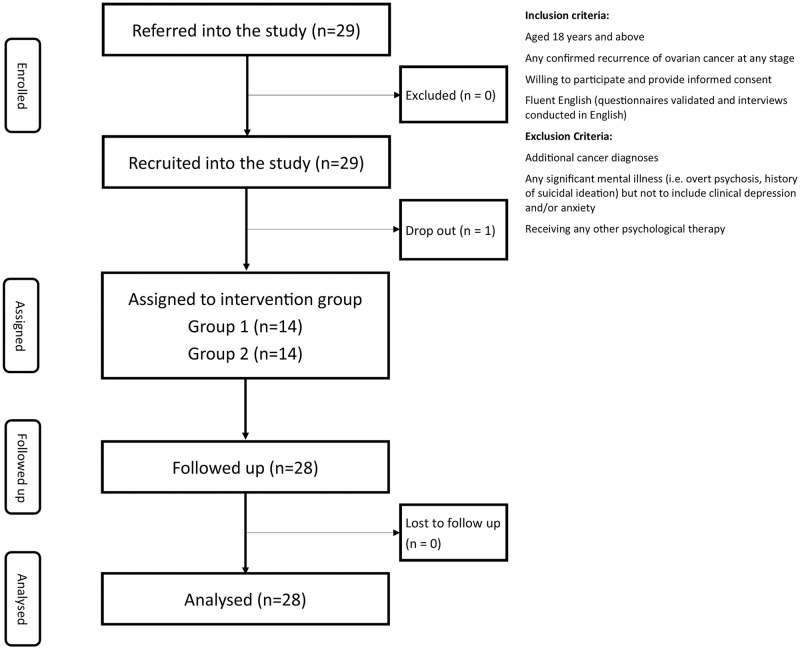
Patient flow diagram.

Eligible participants included women aged 18 years or older with a biopsy-confirmed diagnosis of ovarian cancer who have experienced disease recurrence, at any stage, following initial treatment; were fluent in English; had no concurrent cancer; had no significant mental illness (other than depression and/or anxiety); and were not receiving other psychological therapy (Figure 1). Eligible patients, irrespective of the time since disease recurrence, were approached about referral to the study by their clinical care teams during outpatient clinics at the single recruitment site. All participants provided written informed consent.

Twenty-eight participants were recruited to the study and split into two groups composed of 14 participants each. The program included six group sessions lasting 1.5 hours each, delivered at weekly intervals. Each group sequentially received an identical intervention, with all sessions facilitated by the same qualified mindfulness teacher and attended by the same specialist cancer nurse. Participants were invited to bring a “buddy” (family member or friend) to accompany them and provide support, if they wished. The program trained in both informal and formal practice, including breath awareness, body scan practice, observing thoughts, exploring difficulties, and cultivating loving kindness, with specific practices on reducing stress and coping with anxiety incorporated (Supplemental Material, available online). Additional relaxation practices and simple mindful movements were included, as well as short mindfulness tips. Each session included short meditations (between 10 and 25 minutes), which were also available electronically as a CD or via download for home practice. Participants were encouraged to keep a daily journal and practice log. A workbook accompanied each session, which participants could also use if they were unable to attend a session. All other clinical care continued as normal.

Assessments were conducted prior to starting the intervention, then at baseline (immediately preintervention), six weeks (immediately postintervention), and 12 weeks. Sociodemographic data were collected from a brief self-reported questionnaire. Outcome data were collected via postal self-administered written questionnaires, and biomarker analyses of salivary and blood samples were collected during site visits for attendance at the mindfulness sessions.

Three formal focus groups were also conducted with each of the two groups, held six weeks apart (immediately before and after the intervention, and at six weeks after the final session). The focus groups lasted approximately one hour and used both topic guides and group discussion. A pre-intervention focus group was used to understand participants’ knowledge of mindfulness and their motivations and expectations from the sessions; the Week 6 focus group was intended to explore participants’ experiences of the practices, and the final focus group to explore their views and acceptability of the mindfulness intervention in the longer term.

### Outcomes

Four different questionnaires were completed at three time points (baseline, 6 weeks, and 12 weeks from start of intervention). The questionnaires administered were the Hospital Anxiety and Depression Scale (HADS),^25^ the Warwick/Edinburgh Mental Well-being Scale (WEMWBS),^26^ the Freiburg Mindfulness Inventory (FMI),^27^ and the European Organisation for Research and Treatment of Cancer Quality of Life Questionnaire OV28 (EORTC-QLQ-OV28).^28^ The HADS is a standard, validated measure of mood disorder, which was used to identify clinically meaningful changes in depression and anxiety. The WEMWBS is a short and reliable measure of mental well-being composed of positively worded items relating to positive mental health. The FMI is a 30-item scale designed to measure the concept of mindfulness, using self-reporting of mindfulness qualities such as awareness of the present moment, non-judgmental accepting attitude, and openness to negative states. The EORTC-QLQ-OV28 is an internationally recognized tool for measuring disease-specific quality of life in patients with ovarian cancer and includes both functional and symptom scales.

Salivary cortisol levels were performed at three time points (baseline, 6 weeks, and 12 weeks from start of intervention). Cortisol is a glucocorticoid hormone released in response to stress, and salivary levels of cortisol offer a minimally invasive method of assessing physiological stress responses.^29^ Abnormal patterns of secretion have been reported in populations with ovarian cancer, and have been associated with functional disability, fatigue, and depression.^30^ Measurements of both the awakening and diurnal responses were taken as indicators of hypothalamo-pituitary-adrenal axis dysfunction, in line with recognized recommendations,^31^ and participants collected saliva samples on awakening, and at 0.5, 3, 7, and 12 hours after awakening over two consecutive days at each of the three time points. Saliva samples were collected by participants at home using SalivaBio Oral Swabs (Salimetrics LLC, Carlsbad, CA), which were then stored temporarily in their domestic refrigerator before returning to the research team at the subsequent mindfulness session. Samples were then processed by centrifugation and stored at −80°C^32^ until analysis by enzyme-linked immunosorbent assay (ELISA) at a university research laboratory in line with the manufacturer’s instructions.

Blood tumor marker levels were also performed as biomarker tests at two time points (baseline and 12 weeks from start of intervention). Elevated serum levels of the mucin-like glycoprotein cancer antigen 125 (CA-125) are an established indicator of response to treatment and progression or recurrence of disease, and measurement of CA-125 is part of the usual care of ovarian cancer patients.^33^ Venous blood samples (8 mL) were taken prior to routine clinic visits, and then processed by centrifugation and stored at −80°C until analysis. Levels of CA125 were determined by immunoassay at the study site’s NHS Pathology Service.

Qualitative information was collected at six formal focus groups in total across both cohorts, held at baseline (preintervention, n = 2), 6 weeks (n = 3), and 12 weeks (n = 2) from start of the intervention. This was sufficient to achieve saturation. The focus groups were digitally recorded and transcribed verbatim. A thematic analysis^34^ was conducted to fracture and reorganize the data into codes, and iteratively search for themes from the participants’ discussions. Coding was carried out by the first author, a psychologist who had previously conducted research on ovarian cancer, and attended the mindfulness sessions as an observer. Identified themes were discussed with three other authors (GD, who had run the mindfulness sessions; CCY, who had also attended them; and RG) and disagreements resolved by discussion.

### Statistical Analyses

All analyses were carried out using Microsoft Excel. This was a small feasibility study; therefore, analyses have remained descriptive, without inferential testing.

## Results

The study was conducted between November 2016 and June 2017. Of the 29 women referred to the intervention, all 29 were eligible for recruitment to the study and all 29 consented to take part (Figure 1). One patient withdrew from the study early in the program due to non-engagement. The overall program attendance was 89%, and all participants attended at least three of the six sessions; reasons for non-attendance included holidays, illness, and medical treatments. Follow-up questionnaires were returned (although not always fully completed) by all 28 women who remained in the study.

Sociodemographic and clinical characteristics of participants are reported in Table 1.

**Table 1.** Sociodemographic and Clinical Characteristics of the Study Participants.

Characteristic	Intervention Group (N = 28)
Sociodemographic	
Age, years, mean (SD)	59 (10)
Education, n (%)	
Secondary school	7 (25)
College	10 (36)
Undergraduate	5 (18)
Postgraduate	5 (18)
Not answered	1 (3)
Employment, n (%)	
Retired	9 (32)
Full-time employed	7 (25)
Part-time employed	4 (14)
Housewife	4 (14)
Self-employed	1 (3)
Unemployed	3 (11)
Marital status, n (%)	
Married	21 (75)
Cohabiting	1 (3)
Widowed	3 (11)
Single	3 (11)
Clinical	
Time since initial diagnosis, years, mean (SD)	2.76 (1.94)
CA125 level, units/mL, mean (SD)	311 (1059)

The mean scores and standard deviations for the HADS, WEMWBS, FMI, and EORTC-QLQ-OV28 scales at Week 1, 6, and 12 are shown in Table 2.

**Table 2.** Mean Scores for the Main Variables at Weeks 1, 6, and 12.^a^

Outcome	Week 1	Week 6	Week 12
HADS			
Anxiety	9.56 (4.93)	8.38 (4.49)	6.94 (4.84)
Depression	5.46 (3.98)	4.38 (3.43)	2.83 (2.17)
WEMWBS	47 (10)	52 (14)	54 (7)
FMI	32 (10)	38 (7)	40 (7)
EORTC-QLQ-OV28			
Functional			
Body image	53 (31)	69 (29)	59 (28)
Sexuality	80 (21)	75 (40)	72 (33)
Attitude to disease/treatment	40 (30)	51 (29)	54 (32)
Symptom			
Abdominal symptoms	20 (17)	15 (14)	24 (25)
Peripheral neuropathy	30 (29)	26 (30)	27 (28)
Hormonal symptoms	29 (33)	19 (28)	25 (34)
Other chemotherapy side effects	25 (19)	20 (20)	23 (18)
Hair loss	16 (31)	9 (21)	15 (30)

Abbreviations: HADS, Hospital Anxiety and Depression Scale; WEMWBS, Warwick/Edinburgh Mental Well-being Scale; FMI, Freiburg Mindfulness Inventory; EORTC-QLQ-OV28, European Organization for Research and Treatment of Cancer Quality of Life Questionnaire OV28.

a Data are presented as mean (standard deviation) unless otherwise stated.

**Figure 2.**
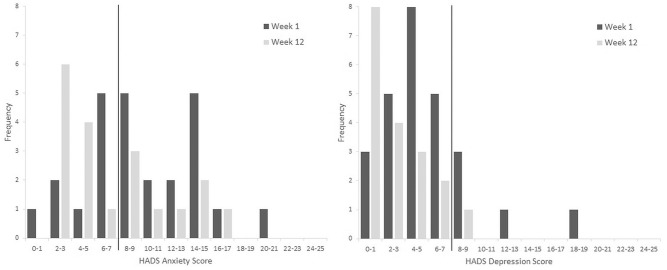
Changes in number of patients with Hospital Anxiety and Depression Scale anxiety and depression scores at Week 1 (black) and Week 12 (gray). Threshold of a score of 8 (black line).

Both the HADS Anxiety and HADS Depression scores show downward trends between baseline and Week 12 of 2.62 and 2.63 points, respectively. A HADS score of 8 to 10 is considered to indicate a mild case in both depression and anxiety, and higher scores indicate more severe symptoms.^35^ Using these cutoffs, 8% of the study group suffered with depression at baseline, and 60% with anxiety. At 6 weeks, the proportion with depression remained unchanged but the proportion with anxiety had fallen to 42%. By 12 weeks, only one participant had a HADS depression score of 8 or more, and 32% had a HADS anxiety score of 8 or more (see Figure 2). However, the percentage of missing data (defined as the percentage of all participants with no reported HADS score) ranged from 6% at baseline to 33% at week 12.

The WEMWBS and FMI scales showed an increase in mean scores between baseline and Week 12 of 7 and 8 points, respectively (Table 2), indicating an improvement in mental well-being and mindfulness, respectively. The mean EORTC-QLQ-OV28 scores showed an initial trend toward improvement for both the functional and symptom subscales at Week 6, except in the sexuality subscale (Table 2). These improvements are not sustained at Week 12, although all mean EORTC-QLQ-OV28 scores (with the exception of abdominal symptoms) remained below the baseline values.

**Figure 3.**
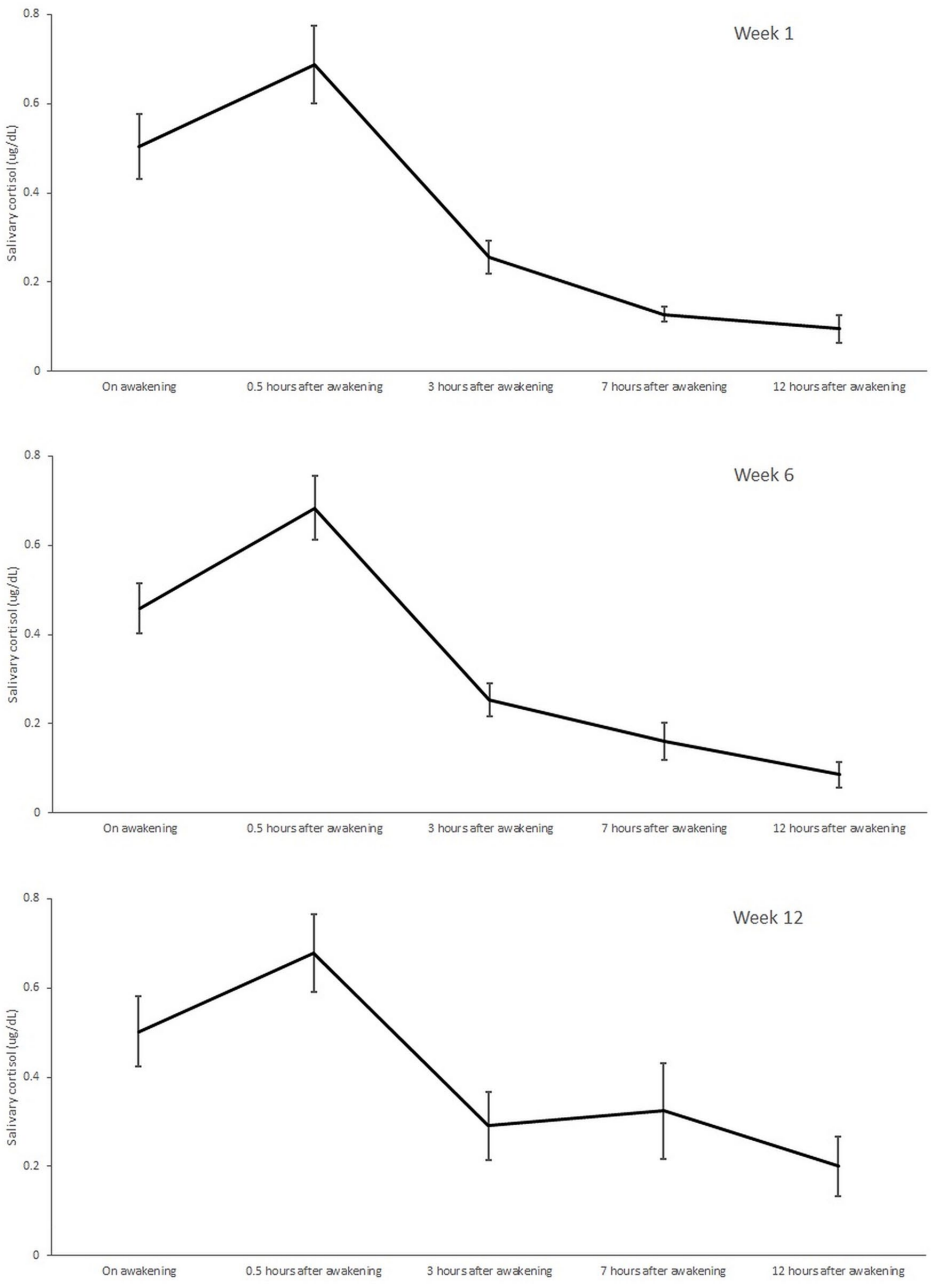
Changes in diurnal cortisol levels between Weeks 1, 6, and 12 including standard error about the mean (vertical bars).

Cortisol levels are presented in Table 3 and showed no difference in average daily mean values from baseline at either Week 6 or Week 12. However, the mean daily values are affected by high variability over the course of the day; therefore, the mean cortisol levels at each of the five collection time points were also analyzed (Table 3). The variation in cortisol levels are shown graphically in Figure 3 and demonstrate “normal” diurnal variation as classified by Touitou et al,^36^ with a clear rise post-awakening to a peak at 0.5 hours and a gradual continuous decline thereafter during the day (Figure 3). The mean variations in diurnal levels of cortisol again demonstrated no meaningful changes following the mindfulness intervention at any of the collection time points assessed. The dynamic change in cortisol following awakening was analyzed by calculating the difference in levels between waking and 30 minutes after waking,^37^ and also showed little difference in patterns of post-awakening cortisol secretion (Table 3). However, salivary samples were poorly collected, and missing data (defined as the percentage of all participants with no reported cortisol level) ranged from 13% of participants at Week 1 to 40% at Week 12.

**Table 3.** Mean Cortisol Levels for All Participants as a Daily Average, at Each of the Five Collection Times and as an Awakening Response, for Weeks 1, 6, and 12.^a^

Cortisol Level (µg/mL)	Week 1	Week 6	Week 12
Daily mean cortisol level	0.34 (0.35)	0.33 (0.31)	0.34 (0.33)
Mean cortisol on awakening	0.51 (0.37)	0.46 (0.27)	0.50 (0.35)
Mean cortisol 0.5 hours after waking	0.69 (0.44)	0.68 (0.35)	0.68 (0.37)
Mean cortisol 3 hours after waking	0.26 (0.19)	0.25 (0.18)	0.29 (0.33)
Mean cortisol 7 hours after waking	0.13 (0.08)	0.16 (0.20)	0.32 (0.46)
Mean cortisol 12 hours after waking	0.10 (0.16)	0.09 (0.14)	0.20 (0.29)
Mean cortisol awakening response	0.18 (0.29)	0.23 (0.23)	0.18 (0.28)

a Data are presented as mean (standard deviation).

There was no change in CA125 values over the study period. Plasma levels of CA125 varied widely between participants at both Week 1 and Week 12, with calculated medians of 16 units/mL (interquartile range [IQR] = 53) at baseline and 23 units/mL (IQR = 166) after the mindfulness intervention.

Qualitative findings relating to the feasibility and acceptability of the mindfulness sessions identified three main themes from the three focus group sessions held.

### Experience of Mindfulness Sessions and Practice

Participants liked the group-based format, as they felt supported by a connection with other participants and motivated by the progress they saw in fellow participants. They felt a connection with others in a similar situation, with whom they could talk about both the techniques and their experiences.

I think it’s as much meeting other people in the same situation. (P5, cohort 2, final focus group)

Many felt more relaxed as the program progressed and appreciated the facilitator.

[She was] very approachable and friendly. . . . You could tell she was passionate about it. (P1, cohort 2, 6-week focus group)

Mindfulness group sessions enabled them to develop both a skill and a support network, which they felt would be hard to achieve if the intervention was delivered remotely, for example, online.

Participants were very positive about the experience of practicing mindfulness, and several reported that it also helped their partners. They saw practicing mindfulness as an opportunity to take time from their daily routines, to be kind to themselves and prioritize their bodies, and felt it made them take stock and focus on the positives.

I think I’m kinder to myself as a result of it, because I feel as though I’m taking time out for me to replenish. (P3, cohort 2, final focus group)

However, participants reported that some aspects of the program took more getting used to. Some found it information-heavy, although the information was considered extremely useful, and felt that it took them about three or four weeks to connect with the mindfulness.

. . . we had a new thing every week—it would have been nice to have gone back and revisited a couple of techniques. (P5, cohort 1, final focus group)

The sessions involved a lot of time sitting, and participants would have preferred a short break in the middle, and some standing mindfulness every week.

Being stood up meant. . . . I personally feel like I have a lot more energy this week and we’ve had a lot more conversation . . . with movement each week or a chance to have a break—it just sort of helps. (P1, cohort 2, week 6 focus group)

### Benefits and Difficulties of Mindfulness

Participants saw mindfulness as a tool to help them focus and cope with stress. They felt it changed their way of thinking and reported using it when negative thoughts came into their head, or in specific stressful situations, such as scans, blood tests, and hospital appointments:

It’s given you a tool to use, you know, so you’ve got it there all the time, which you know that if you don’t feel 100%, you can think about that and it helps you calm down. (P4, cohort 1, final focus group)

I found it extremely useful, even lying in that CT scanner, because you’re lying there, the pictures are being taken and you think . . . what are they going to find. And then suddenly the exercise came into my mind and I’m lying there and I’m closing my eyes and I’m thinking what I can smell, what I can hear, I can taste, and I’m concentrating on my breathing and it just got me through the scan. (P2, cohort 1, week 6 focus group)

Participants noticed a number of mental health benefits, including feeling calmer and more relaxed, being more positive, and feeling less anxious and panicky.

Participants felt that many of the different practices had specific benefits. For example, all participants reported sleeping better, with particular reference to using the Progressive Neuromuscular Relaxation at night, whereas the Emergency Mind Aid was seen as a tool for stopping events from spiraling out of control.

I wake up in the middle of the night. . . . I’ll sometimes do, you know, still lying in bed, tensing muscles gradually and releasing them, just drift off back to sleep. (P5, cohort 2, final focus group)

Applying mindfulness to everyday activities was felt to increase concentration throughout the day.

Despite an overall positive view of mindfulness, participants experienced some difficulties with specific practices. Many participants reported being unused to sitting still and some found it hard to concentrate. This was expected, as mindfulness practice raises awareness of being unable to concentrate. However, the most significant issue mentioned by almost all participants was the negative emotions that came up during the “Exploring Difficulties” practice, when they did it in the session, particularly if they were currently feeling positive.

If you’re in a good place, the last thing you want to do is stop and think of something that’s troubling you. (P4, cohort 2, week 6 focus group)

However, they were able to understand the rationale behind the “Exploring Difficulties” practice, which they saw as something to draw on if they were experiencing an all-consuming negative situation.

I can see the positive in learning to do it, but it would take some summoning of courage to perhaps redo it. (P6, cohort 2, week 6 focus group)

### Barriers and Facilitators to Practicing Mindfulness

Support from others seemed to be an important factor in enabling practice. Some women found practicing together with partners or friends was helpful. Others saw being committed to a specific goal, or having a schedule, as important for completing home practice.

I made an arrangement with somebody else that I would text them it was done and they would then text—nag me if I hadn’t done it. (P3, second cohort, final focus group)

Physical aids also facilitated practice. Participants found it beneficial to practice with the CD, and many had downloaded the practices to their phones or tablets.

I’ve got it on my phone so when we go to bed, press it, listen to it, and it turns itself off at the end. (Participant’s partner, cohort 2, final focus group)

However, participants who were working full time said that they found it hard to make the time to practice, and some reported distractions at home could be problematic.

## Discussion

This study has demonstrated the feasibility of delivering a mindfulness-based intervention to women with recurrent ovarian cancer within a standard cancer care pathway in a UK hospital setting. We observed high recruitment and retention rates, and participants told us that they found the program acceptable. Mindfulness was considered by participants to be a useful tool when managing difficult experiences, and the outcomes suggest a positive impact on depression and anxiety symptoms, mental well-being and mindfulness, and health-related quality of life. Development of this study may consider introducing screening for clinical levels of anxiety or depression at baseline for entry into future trials, to enable clearer analysis of any improvement following the mindfulness intervention.

These preliminary results did not, however, suggest any effect on the physiological markers studied (salivary cortisol profiles or CA125 biomarker levels). Cortisol levels in particular were challenging to monitor; swabs were not always taken consistently, correctly or sufficiently, and participants found taking samples inconvenient and burdensome. The evidence of mindfulness effects on cortisol levels is mixed,^38^ and the insights from this study may be impacted by the relatively “normal” baseline cortisol profiles of our participant population.^36^ Future development of this protocol, therefore, may review the feasibility of some of the outcome measures, and consider alternative means of sample collection or measurement, and/or approaches to simulation of the potential impact of missing data during sample size calculations.

Participants’ experiences and perceptions were key to the findings of this study, and their feedback identified important areas for further study. Participants told us that the social support network resulting from the program was important to them, and many were still in touch with each other after the study had ended. This corresponds with the findings of similar studies,^39^ and it suggests that future work should investigate the impact of this social interaction on the effectiveness of the intervention. More detailed study is also needed into the impact of mindfulness on sleep, as improved sleep duration and patterns were another strong theme from participants’ feedback.

This work sought to test the study protocol and operational feasibility and acceptability of this intervention, to help design further confirmatory studies; accordingly, it includes an appropriately small sample size without a control group. The findings, therefore, require cautious interpretation given the size, design, and duration of the study. There is a risk of bias and imprecision due to missing data, particularly at Week 12, and development of the protocol should address challenges in data collection at this time point. We additionally note other factors that may have influenced the success of this work. The gender and age of the study population may have meant that they had more time to participate in mindfulness activities and were more receptive to this type of intervention, and there is a risk of influence by the “popularity effect” of a growing acceptance of mindfulness techniques.^40^ The mindfulness intervention delivered during the study differed from standard mindfulness-based stress reduction programs, being a reduced program, with fewer, shorter sessions and practices. The program also stated explicitly the possible benefits of practice and offered tools that participants could use in certain situations; this differs from the transitional programs of self-discovery offered by conventional mindfulness. Future studies should investigate whether mindfulness in general, or specific interventional approaches, are most effective for a given illness.

## Supplemental Material

REVISED_Supplemental_Material_-_Programme_of_Practice – Supplemental material for Mindfulness-Based Interventions in Recurrent Ovarian Cancer: A Mixed-Methods Feasibility Study Click here for additional data file. Supplemental material, REVISED_Supplemental_Material_-_Programme_of_Practice for Mindfulness-Based Interventions in Recurrent Ovarian Cancer: A Mixed-Methods Feasibility Study by Emily Arden-Close, Felicity Mitchell, Gail Davies, Lauren Bell, Carole Fogg, Ruth Tarrant, Roslyn Gibbs and Chit Cheng Yeoh in Integrative Cancer Therapies
