# Supplementary material for: Mindfulness-Based Interventions in Recurrent Ovarian Cancer: A Mixed-Methods Feasibility Study
Source: Integr Cancer Ther. 2020 Mar 16;19:1534735420908341. doi: 10.1177/1534735420908341 (PMC7076576; doi:10.1177/1534735420908341)
Supplement: REVISED_Supplemental_Material_-_Programme_of_Practice – Supplemental material for Mindfulness-Based Interventions in Recurrent Ovarian Cancer: A Mixed-Methods Feasibility Study [file REVISED_Supplemental_Material_-_Programme_of_Practice.pdf]

Supplementary Information 1

Detailed Overview of the Mindfulness-Based Intervention in Recurrent Ovarian Cancer

This programme was delivered as six sessions, developed and delivered by an experienced and qualified instructor complying with UK good practice guidelines

**Session 1**	**Checking in and releasing tension**<br>Commonly, when we are anxious or stressed, we hold tension in the body. Learning to "check in" and "release tension" is a useful way to reduce stress levels. We will use this simple informal practice to consciously switch on the parasympathetic nervous system. Over time, it is possible to identify what experiences or thoughts cause us to tense and hold our breath, and to deliberately let them go.
**Theme & content**	**Getting started on a personal mindfulness journey**<br>*Mindfulness is about starting from where you are. It is experiential and this journey will start with these women sharing their own stories so far, their hopes and fears about the future.*<br>Introductions<br>• Personal stories & what they hope to gain from this course<br>• Commitment to practice<br>Ground rules of the group & safety measures/support<br>Looking after yourself during the course.<br>Introduction to mindfulness<br>• What it is?<br>• Relevance to participants<br>• Short video of how others have used mindfulness to deal with cancer & survivorship<br>How mindfulness works<br>• Intention (Set this in Journal as a motivation to practice)<br>• Attention & Approach<br>• Discuss the importance of approach – non-judging, kindness, curiosity etc…<br>Stress<br>• Modern living & extra burden of cancer<br>• Biochemical impact<br>• Fine tuning the autonomic system<br>• How to switch on the relaxation response<br>Befriending the breath<br>• Power of the breath<br>• Practical issues around breathing properly<br>• Using the breath to relax or invigorate
**Practices**	Informal mindfulness practice – "checking in and releasing tension"
**Teaching points**	Learning to release and let go of tension<br>Relaxation is the natural state
**Homework**	Daily practice of "checking in and releasing tension"<br>Practice log & journal (observations – thoughts, feelings, body sensations, comments)<br>Choose specific approach/ attitude each day and explore that as you go about activities

**Session 2**	**Training attention using a Breath Awareness Practice**<br>Learning to pay attention, noticing when the mind has wandered and simply bringing it back is central to mindfulness (Gunaratana, 2002). Mind-wandering is prevalent, is associated with being unhappy and is exacerbated by stress (Killingsworth & Gilbert, 2010). It is known to correlate with neural activity in a network of brain areas that support self-referential processing, known as the default-mode network (Brewer *et al.* 2011; Farb *et al.* 2007). Consequently, we can easily lose conscious control; amplify emotions and fall into habitual patterns of thoughts and behaviours, so exacerbating our stresses, anxieties and sadness (Williams & Penman 2011). Furthermore, it is thought that concentration-based mindfulness meditation helps "de-automise" mental processes that cloud perception of stimuli in the present moment (Lutz *et al.* 2008) and that this is central to the benefits of practising mindfulness, coming off automatic pilot and seeing things clearly as they are.<br>Typically, the breath - sensations of breathing - is used to train attention (Grossman, 2010; Gunaratana, 2002). This provides an anchor to the present moment and facilitates disengaging with other mind activities such as rumination (Williams *et al.* 2007). As the mind becomes focused it has a calming effect, reducing stress and anxiety.
**Theme & content**	**"Gathering a scattered mind"**<br>*We know that nearly 50% of the time our mind is not where we intended it to be and when we are stressed, anxious or have low mood this increases. Furthermore, a wandering mind is not a happy mind; it has a negative bias, and can take us to dark places, caught up in rumination and proliferation. In this session we will use mindfulness to gather and focus a wandering mind; bring it back to a neutral place grounded in the present moment.*<br>"Checking in"<br>• Practice<br>• Discussion of how the week has been - was it possible to practice?<br>• Experiences of mindfulness able to share<br>• Barriers and obstacles to practice<br>Living on autopilot & the dangers of a wandering mind<br>• Background of mind wandering and where it can take us<br>• Common having survived cancer that there are anxieties, worries and how mindfulness can be used to gather the mind when it is all over the place and bring it back to focus on something neutral such as the breath<br>• Taking control of a wandering mind<br>• Video showing impact of mindfulness on the brain<br>Training attention<br>• Breath awareness<br>• Breath used as focus of attention – it is in the present moment and considered a neutral focus<br>• Reminder that this is not just a concentration exercise as the attitude we bring is important e.g. kindness and curiosity<br>• If problems with the breath provoking anxiety, can use external object such as sound<br>• Experience of the practice discussed in pairs and then open to the group<br>Revisit Breath and breathing from first session<br>• Power of the breath – indicator of how we are

	• Practices to invigorate or relax<br>• Now introduced as an anchor to gather and calm a scattered mind.<br>What causes you to hold your breath?<br>• Notice when this happens, release and let go
**Practices**	Informal mindfulness practice – "checking in and releasing tension"<br>Formal attention training using Breath Awareness
**Teaching points**	Learning to pay attention, beginning to gain more control over a wandering mind by focusing it on a neutral object such as the breath (or sound). As we focus the mind, it calms the mind.<br>The Breath awareness practice is used to skilfully attend to mind-wandering, starting to allow things to be as they are. Learning how the breath can be a "vehicle" for directing awareness.<br>This is used in all other practices (Killingsworth & Gilbert, 2010)
**Homework**	Daily Formal practice of attention training using Breath Awareness<br>Informal mindfulness practice – "checking in and releasing tension"<br>Practice log & journal (observations – thoughts, feelings, body sensations, comments)<br>Choose specific approach/attitude each day and explore that as you go about activities

**Session 3**	**Session 3: Reconnecting mind and body using the Body Scan Practice**<br>Practices, such as the body scan, use body sensations as the focus of attention (Dreeben *et al.* 2013). Tuning into body sensations cultivates an awareness of the body-mind system. Formal practice has a significant effect on the insula (Hölzel *et al.* 2008; Lazar *et al.* 2005), which is implicated in interoceptive perception (Craig, 2003). This improvement in interoceptive perception, provides valuable information about the general state of our bodies, our stress levels, our moods and our behavioural urges (Fletcher *et al.* 2010). Effectively, through practice we are training to use the body as a barometer (Williams & Penman, 2011). Over time, we are able to identify personal signatures of emotions such as stress and anxiety and we can use these physiological signals from the body as an early warning system to offset emotional hijacks (Williams & Penman, 2011).<br>The body scan is used to train familiarity of body sensations as they arise, learning to approach and explore them, effectively reducing avoidance of contacting them. This is an important therapeutic process, which is useful in conditions such as panic disorder, some form of posttraumatic stress disorder and pain (Fletcher *et al.* 2010).
**Theme & content**	**"Reconnecting mind & body"**<br>*Emotions are useful physiological signals unfortunately we often don't "read" them correctly and amplify them to the extent that they are overwhelming. We are subject to "emotional hijack" and they seem to be more in control of us rather than the other way around.*<br>*Using mindfulness we can increase awareness of body sensations and begin to use the body as a barometer, an early warning system for emotions arising and gain more emotional control.*<br>"Checking in"<br>• Practice<br>• Discussion of how the week has been - was it possible to practice?<br>• Experiences of mindfulness able to share<br>• Barriers and obstacles to practice<br>Emotions

<table>
<tr><td></td><td>What are they, why are they useful?
Interconnectedness – body – mind loop
Body as amplifier or early warning detection system
Examples of embodied cognition
Physiological signatures in the body
Exercise – bringing fear or happiness to mind, what does it feel like in the body?
Video – Bodily maps of emotion
Practice Body Scan
Training attention broad and narrow using sensations in the body
Increasing awareness of bodily sensations
Appreciating the difference between thinking about sensations and experiencing them
Body is a different way of processing emotions (later session)
If brings up difficulties (may be hard to “befriend” the body) then returning to the breath as an anchor.
Experience of the practice discussed in pairs and then open to the group
Emotional regulation
Emotions detected as physiological signals are quick and tangible
Emotions drive behaviour and colour experience
Refractory period
How mindfulness by increasing self-awareness can help
Simple Tool when caught up in emotion e.g. RAIN</td></tr>
<tr><td>Practices</td><td>Informal mindfulness practice – “checking in and releasing tension”
Body Scan</td></tr>
<tr><td>Teaching points</td><td>Reconnecting mind and body. Increasing body awareness.
Identifying own physiological signatures of emotions arising and learning to interrupt them over time.
“Body–mind loop”- bringing awareness to body can interrupt this and can be a different way of processing emotions
Using the body as a valuable source of information – bodily maps of emotions (Nummenmaa et al. 2013)
Recognising signals from your body, a personal “barometer” signalling inclement emotions, such as unhappiness, anxiety and stress before they arise (Williams and Penman, 2011)</td></tr>
<tr><td>Homework</td><td>Daily Formal practice of Body scan
Informal mindfulness practice – “checking in and releasing tension”
Practice log & journal (observations – thoughts, feelings, body sensations, comments)
Herbert Benson stress trigger form</td></tr>
</table>

<table>
<tr><td>Session 4</td><td>Session 4: Increasing cognitive awareness using a practice of Breath Body Sounds and Thoughts
A formal meditation practice will be used to guide through observing sensations of breathing, bodily sensations, sounds and thoughts as they come and go without getting caught up in them. In addition, to this practice we will use psycho-education exercises reinforcing that thoughts are just thoughts and not facts (Segal et al. 2013). Opening up to observe thoughts provides an opportunity to better understand how our mind works and how it affects us (Grossman, 2010).</td></tr>
</table>

**Theme & content**	**“Mind games”**<br>*Mindfulness can be used to increase cognitive awareness, to look at what goes on in our heads, the familiar stories and patterns of thoughts. Aware that thoughts are just thoughts and are not facts.*<br>*Appreciating that it is not our experience per se that causes emotions to arise and illicit certain behaviours but rather our interpretation of experience. Using mindfulness to strip away the layers and see things clearly for what they are not what we think they are.*<br>“Checking in”<br>• Practice<br>• Discussion of how the week has been - was it possible to practice?<br>• Experiences of mindfulness able to share<br>• Barriers and obstacles to practice<br>• What is working and not working<br>Exercise – thoughts are not facts<br>• Using simple scenario see where our mind takes us<br>Mind games<br>• Common mental habits<br>• How many do you do?<br>Exercise – Interpretation<br>• Using scenario looking at thoughts & feelings<br>• Aware that we all interpret same thing differently and this impacts on how we feel and what action we want to take<br>Practice – Breath Body Sounds & thoughts<br>• Realising that everything comes and goes<br>• Thoughts come and go<br>• Learning to observe them<br>• Coming back to the breath if caught up or too difficult<br>• Gaining insight into what goes on in our heads<br>• Experience of the practice discussed in pairs and then open to the group<br>Practical ways of dealing with thoughts<br>• Observing rather than pushing away or trying to stop them<br>• Appreciate thoughts are just thoughts not facts – perhaps reappraise<br>Tools for getting out of our heads<br>• Anchor to the breath<br>• Senses – Overthinking mode of mind vs experiential mind<br>• Looking after yourself during the course.<br>Breathing Space<br>• Giving some distance - reappraise<br>Stress<br>• Modern living & extra burden of cancer<br>• Biochemical impact<br>• Fine tuning the autonomic system<br>• How to switch on the relaxation response<br>Befriending the breath

	• Power of the breath<br>• Practical issues around breathing properly<br>• Using the breath to relax or invigorate
**Practices**	Informal mindfulness practice – "checking in and releasing tension"<br>Formal practice – Breath Body Sounds and Thoughts<br>Informal Breathing Space
**Teaching points**	The key learning here is "de-centring" seeing thoughts and feelings simply as thoughts and feelings, as an observer rather than defining ourselves by them. It enables us to step back and effectively be able to relate to them rather than from them, caught up in them. We are starting to cultivate a spaciousness of mind, a wider perspective and so reducing the power of thoughts, able to see "thoughts as thoughts" and not facts. This enables us to see things more clearly as they really are and make better choices.<br>Comparisons are seen between thoughts and sounds; they come and go and they are not "us". Over time we begin to recognise unhelpful thought patterns and learn to read and acknowledge them. They could simply signify a "weather pattern" and serve as a warning.
**Homework**	Daily Formal practice of Breath, Body, Sounds and Thoughts<br>Informal mindfulness practice – "checking in and releasing tension"<br>Informal practice –Breathing Space

**Session 5**	**Session 5: Practices looking at Facing difficulties & cultivating Loving Kindness**<br>Formal mindfulness meditation practices to explicitly explore dealing with difficulties or pain, both physical and emotional, will be explored (Segal *et al.* 2013). Instead of getting caught up in the psychological and emotional struggle with pain and difficulties, individuals are encouraged to live alongside them, employing a more acceptance-based approach (Cusens *et al.* 2010). Acceptance and self-awareness are generally proposed as the mediating factors to explain how mindfulness affects pain (Baer, 2003; Shapiro *et al.* 2006).<br>Harsh self-criticism is key to a wide range of mental health problems, especially depression. Through mindfulness practice kindness and friendliness can be developed, which is more supportive of healthy emotional processing and wellbeing. Kindness is transformative: this change in attitude enhances openness, creativity and happiness, while acting as an antidote to fear and guilt and reducing stress and anxiety (Williams and Penman, 2011). It helps to switch off the "aversion" pathways in the mind, which lead to exhaustion and chronic discontent and rather switching on the "approach" pathways. A formal Loving Kindness practice will be used to cultivate kindness.
**Theme & content**	**"Facing difficulties"**<br>*Dissatisfaction and "suffering" come from wanting things to be different. When we brace against things, push things away, we expend effort and add layers to our suffering.*<br>*Mindfulness offers a different approach, a "softening" and turning towards. This acceptance and letting be allows us to deal with difficulties. Through mindfulness we can put processes into place to manage difficulties adaptively.*<br>*Cultivating kindness and compassion is central to transformation in mindfulness.*<br>"Checking in"<br>• Practice<br>• Discussion of how the week has been - was it possible to practice?

	• Experiences of mindfulness able to share<br>• Barriers and obstacles to practice<br>• What is working and not working?<br>• Have there been any shifts?<br>Facing difficulties – pain physical and emotional<br>• Usual strategies<br>• Using mindfulness – turning towards, acceptance, letting be, curiosity<br>• Reminder of the practice "checking in and releasing tension"<br>Practice – Exploring difficulties<br>• Breath, Body, opening up to intense sensations<br>• Exploring, softening<br>• Bringing difficulty to mind, body sensations, processing emotions in the body<br>• Experience of the practice discussed in pairs and then open to the group<br>Loving kindness – Self compassion<br>• Cultivating kindness and equanimity towards self<br>• Short practice of Loving kindness<br>• Short Self compassion practice as a tool
**Practices**	Formal practice – Exploring difficulties<br>Formal practice – Loving kindness<br>Informal mindfulness practice – "checking in and releasing tension"
**Teaching point**	During the exploring difficulties practice we learn to "drop into" physical sensations in the body when there are strong negative thoughts and emotions around. Turning to these physical sensations helps stop mental proliferation and anchors awareness in the flux of present moment experience. We notice the natural variation, changes in intensity, and it provides one way of "riding the waves" of powerful internal experiences. In doing so, we are able to let go of fuelling them by trying to suppress or change things.
**Homework**	Daily Formal practice of Exploring difficulties or Loving Kindness<br>Informal mindfulness practice – "checking in and releasing tension"<br>Informal practice –Breathing Space

**Session 6**	**Session 6: Formal sitting practice of "choiceless" awareness**<br>As the course progresses and with practice, it becomes possible to move participants to meditate on whatever arises (Lutz *et al.* 2008). Over time, with continued mindfulness practice, participants will become more self-aware, which leads to better self-regulation of cognitions, emotions and behaviours (Gross, 1998).
**Theme & content**	**"Closing and going forward"**<br>*What are my insights from this journey so far and where do I want to go? How do I continue my mindfulness journey in a way that serves me?*<br>"Checking in"<br>• Practice

	• Discussion of how the week has been - was it possible to practice?<br>• Experiences of mindfulness able to share<br>• Barriers and obstacles to practice<br>• What is working and not working?<br>• Have there been any shifts?<br>Recap of the practices & learning so far<br>• Overview of course<br>• Discuss in pairs what you take away<br>• Open to group<br>Formal Practice of Open monitoring or “choiceless” awareness<br>• Awareness of whatever arises<br>• Sitting meditation<br>• Experience of the practice discussed in pairs and then open to the group<br>Cultivating mindfulness in everyday life<br>• Practice of mindful walking or movement<br>• Suggestions of integrating mindfulness<br>Looking after yourself<br>• Nourishing and depleting exercise<br>• Exhaustion funnel<br>• Pacing<br>• Breathing Space with an action step<br>• Gratitude practice<br>Going forward<br>• Open discussion<br>Closing practice
**Practices**	Formal practice of “choiceless” awareness<br>Informal mindfulness practice – “checking in and releasing tension”<br>Mindful movement
**Teaching points**	This practice of “choiceless” awareness focuses on the full range of experience and for many is considered as the formal sitting meditation of mindfulness. It cultivates acceptance of what is by simply allowing whatever arises to be our experience in this moment. Equally accepting pleasant, unpleasant and neutral. Developing a spacious quality of mind, aware of all experiences as they arise and pass away in consciousness. Recognising recurring patterns of the mind. Seeing more deeply into the nature of human experience and life. The experience of “choiceless” awareness practice parallels daily life. As we learn to notice and be with the flux in formal practice, we can bring greater awareness to flux in our daily experience.
**Homework**	Ongoing practice formal and informal, whatever works for you

References

Baer R.A. (2003). Mindfulness Training as a Clinical Intervention: A Conceptual and Empirical Review. Clin. Psychol. Sci. Pract. 10:2 125-43

Brewer, J.A., P.D. Worhunsky, J.R. Gray, Y.Y. Tang, J. Weber, and H. Kober (2011). Meditation experience is associated with differences in default mode network activity and connectivity. Proceedings of the National Academy of Sciences of the United States of America. 108(50): p. 20254-9.

Craig A.D. (2003). Interoception: the sense of the physiological condition of the body. Current Opinion in Neurobiology 13: 500–505

Cusens B., Duggan G.B., Thorn K. & Burch V. (2010). Evaluation of the Breathworks Mindfulness-Based Pain Management Programme: Effects on Well-being and Multiple Measures of Mindfulness. Clin Psychol Pyschother. 17: 63-78

Dreeben S.J., Mamberg K.H. and Salmon P. (2013). The MBSR body scan in clinical practice. Mindfulness 4: 394-401

Farb, N.A.S., Segal, Z.V., Mayberg, H., Bean, J., McKeon, D., Fatima, Z., and Anderson, A.K. (2007). Attending to the present: Mindfulness meditation reveals distinct neural modes of self-reference. Social Cognitive and Affective Neuroscience, 2, 313–322.

Fletcher B, Schoendorff B and Hayes SC (2010). Searching for mindfulness in the brain: A process-oriented approach to examining the neural correlates of mindfulness. Mindfulness 1:41–63 DOI 10.1007/s12671-010-0006-5

Gross, J. J. (1998). The Emerging Field of Emotion Regulation An Integrative Review. Review of General Psychology, 2, 271-299.

Grossman, P. (2010). Mindfulness for Psychologists: Paying Kind Attention to the Perceptible. Mindfulness 1(2):87-97

Gunaratana H. (2002). Mindfulness in plain English. Boston: Wisdom Publications

Hölzel B.K., Ott U, Gard., Hempel H, Weygandt M., Morgen. and Vaitl D

(2008). Investigation of mindfulness meditation practitioners with voxel-based morphometry. Social Cognitive and Affective Neuroscience. 3: 55-61

Killingsworth M.A. and Gilbert D.T. (2010). A Wandering Mind Is an Unhappy Mind Science Vol. 330 no. 6006 p. 932 DOI: 10.1126/science.1192439 BREVIA

Lazar S., Kerr C., Wasserman R.H., Gray J.R., Greve D. N., Treadway M. T., McGarvey M, Quinn B.T., Dusek J.A., Benson H., Rauch S.L., Moore C.I. and Fisch B. (2005). Meditation experience is associated with increased cortical thickness. NeuroReport. 28 November; Vol. 16, No. 17

Lutz, H.A. Slagter, J.D. Dunne, R.J. Davidson (2008). Attention regulation and monitoring in meditation. Trends in Cognitive Science. 12: 163–169

Nummenmaa L., Glerean E., Hari R. and Hietanen J.K. (2013). Bodily maps of emotions. PNAS DOI: 10.1073/pnas.1321664111/-/DCSupplemental.

Segal Z. V., Williams M. G., & Teasdale J. D. (2013). Mindfulness based cognitive behaviour therapy for depression. (Second Edition) New York: Guildford.

Shapiro S.L., Carlson L.E., Astin J.A. and Freedman B. (2006). Mechanisms of mindfulness. Journal of Clinical Psychology. 62: 373-386

Williams M. and Penman D. (2011). Mindfulness a practical guide to finding peace in a frantic world. London: Piatkus
